# Thiyl radical induced *cis*/*trans* isomerism in double bond containing elastomers†

**DOI:** 10.1039/d3ra04157c

**Published:** 2023-08-10

**Authors:** Anureet Kaur, Julien E. Gautrot, Keizo Akutagawa, Douglas Watson, Alan Bickley, James J. C. Busfield

**Affiliations:** a Queen Mary University of London London UK anureet.kaur@qmul.ac.uk j.gautrot@qmul.ac.uk g.cavalli@qmul.ac.uk k.akutagawa@qmul.ac.uk j.busfield@qmul.ac.uk; b Weir Advanced Research Centre Glasgow UK douglas.watson@mail.weir alan.bickley@mail.weir

## Abstract

This report presents an evaluation of thiyl radical-induced *cis*/*trans* isomerism in double bond-containing elastomers, such as natural, polychloroprene, and polybutadiene rubbers. The study aims to extensively investigate structural changes in polymers after functionalisation using thiol–ene chemistry, a useful click reaction for modifying polymers and developing materials with new functionalities. The paper reports on the use of different thiols, and *cis*/*trans* isomerism was detected through ^1^H NMR analysis, even at very low alkene/thiol mole ratios. The study finds that the configurational arrangements between non-functionalised elastomer units and thiolated units followed a *trans*-functionalised-*cis* units arrangement up to an alkene/thiol mole feed ratio of 0.3, while from 0.4 onward, a combination of *trans*-functionalised-*cis* and *cis*-functionalised-*trans* configurations are found. Additionally, it is observed that by increasing the level of functionalisation, the glass transition temperature of the resulting modified elastomer also increases. Overall, this study provides valuable insights into the effects of thiol–ene chemistry on the structure and properties of elastomers and could have important implications for the development of new materials with enhanced functionality.

## Abstract 2

This report presents an evaluation of thiyl radical-induced *cis*/*trans* isomerism in double bond-containing elastomers, such as natural, polychloroprene, and polybutadiene rubbers.

## Graphical abstract

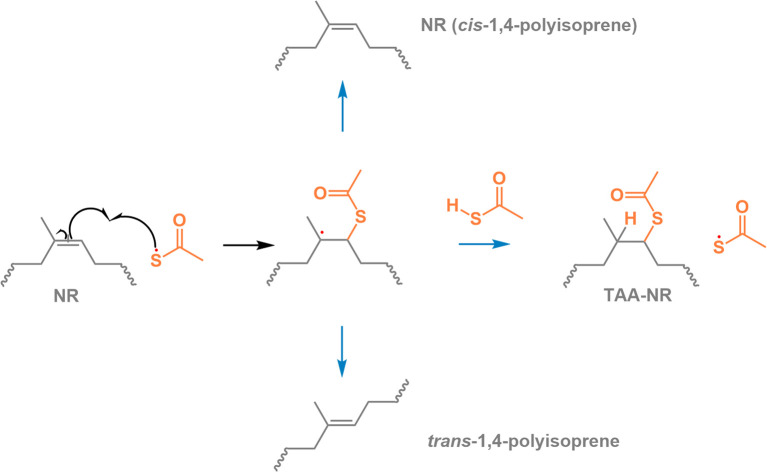

## 1 Introduction

The thiol–ene reaction, also known as alkene hydrothiolation, is a reaction between a thiol and an alkene to give as the final product a thioether containing C–S–C as a functional group. The reaction was first discussed by Theodor Posner in 1905 where in the attempt to investigate an eventual condensation of the carboxyl unit of unsaturated ketones with mercaptans he discovered that the double bond always added a molecule of mercaptan with great ease, concluding that the addition occurred regardless of the proximity of a ketone group.^1^ The number of reports implementing the addition of thiols to various substrates increased together with the increasing importance of the thiol functionality in organic chemistry leading to the publication of The Chemistry of the Thiol Group in 1974 covering topics like structure configuration, synthesis, reactivity, isomerism, and application in photochemistry and thermochemistry.^2^ A large amount of literature has reported on different aspects of thiol–ene chemistry, such as photopolymerization^3–5^ and crosslinking,^6,7^ polymer modification,^4,5,8^ polymer coupling^8^ and development of novel biomaterials^9–12^ and hydrogels.^13,14^ From a reactivity point of view several reports investigated the influence of the catalytic mechanism,^15^ the alkene and thiol molecular architecture and environment^13,16^ and on the solvent effect.^17^ From a final application point of view an interesting review has been published by Sticker *et al.* discussing the benefits of thiol–ene based polymers in microfluidic devices for on-chip assays and chemical analysis.^18^ These reviews refer to the thiol–ene reaction as “click reaction” and “anti-Markovnikov addition”. In 2001 Kolb *et al.* defined a set of criteria to classify a type of reaction as a click reaction: modular reaction, wide in scope, giving high yields and a stable product, stereospecific, ideally non sensitive to oxygen and water, that can occur in an easily removable solvent, rely on readily available starting materials and reagents and give inoffensive by-products that can be removed by simple purification methods such as crystallization, precipitation and distillation.^19^ Thiol–ene chemistry has been used as a powerful tool to develop new polymer materials, where new properties are achieved depending on the final applications needs. In particular, it has been used in order to introduce alternative ways of crosslinking,^20–33^ for reversible cross-linking,^34,35^ for self-healing purposes,^36–39^ for surface functionalisation^40–49^ and enhance blend compatibility,^50–55^ on double bond containing elastomers such as Natural Rubber (NR), Synthetic Polyisoprene (IR), Polybutadiene Rubber (BR), Nitrile Rubber (NBR), Styrene–Butadiene Rubber (SBR) and Ethylene Propylene Diene Monomer Rubber (EPDM). More recently, thiol–ene reaction was found to be effective also on low reactivity elastomers, such as Polychloroprene Rubber (CR), to develop a recyclable and self-healable material.^56^ Thiyl radicals are known to induce *cis*/*trans* isomerism in di-substituted carbon–carbon double bonds. This effect was observed in fatty acid molecules such as oleic acid^57^ and arachidonic acid,^58^ but it has not yet been fully explored for radical thiol–ene chemistry applied to double bond containing elastomers. NR is made of repeated *cis*-1,4-polyisoprene units, which can form crystalline domains within the matrix over time, in particular when the rubber is not vulcanised or at low temperatures. Cross-linked NR, on the other hand, cannot readily crystallise by itself. However, in 1925 Katz *et al.* discovered that vulcanised NR can crystallise under strain and defined the phenomena as strain-induced crystallisation (SIC).^59^ Since then, studies have been carried to understand this phenomenon^60^ which is responsible for enhancing mechanical properties,^61^ such as crack growth behaviour^62–66^ and fatigue behaviour.^67–69^ SIC in NR occurs at a rate depending on the bulk viscosity of the matrix, strictly dependent to the isomer configuration arrangement:^70,71^ the formation of *trans* isomers would change the bulk viscosity of the matrix, which would lead to a change in the rate at which chains can rearrange, inevitably impacting this SIC property. In order to establish how cross-linking and modification of natural rubber and related olefinic polymers *via* thiol–ene chemistry impacts on the mechanical properties of these materials, their self-healing and recyclability, it is important to consider the impact of this chemistry on the chemical structure of these materials.

In the present work, *cis*/*trans* isomerism occurring with NR, *cis*-BR and CR (mainly *trans* units) in response to the influence of thiyl radicals generated from different thiols has been studied *via* dynamic differential scanning calorimetry, gel permeation chromatography and nuclear magnetic resonance spectroscopy. The characterisation analysis was then used to find correlations between the observed isomerism and their thermodynamic properties. Additional analysis was also carried to investigate the mechanism of isomerisation to see if it could be activated in presence of thiols or also with carbon radicals.

## 2 Experimental section

### 2.1 Materials

Natural rubber (NR; SMR CV60 grade) was purchased from Tun Abdul Razak Research Centre. Chloroprene rubber (CR; SN 122 grade) was supplied by The Weir Group PLC. Polybutadiene rubber (BR; 98% *cis*, average *M*_w_ 200 000–300 000), thioacetic acid (TAA; ≥96% purity), thioglycolic acid (TGA; ≥99% purity), 2-propanethiol (PPT; ≥97.0% purity, GC grade), 2-mercaptoethanol (BME; ≥99.0% purity), 2,2′-azobis(2-methylpropionitrile) (AIBN; 98% purity), 2,2-dimethoxy-2-phenylacetophenone (DMPA, 99% purity), toluene (≥99.9% purity, HPLC grade), dichloromethane (≥99.9% purity, ACS reagent), methanol (≥99.9% purity, HPLC grade) and chloroform-d (CDCl_3_; 99.8 atom% D) were all purchased from Sigma-Aldrich Co Ltd.

### 2.2 Synthesis procedures

#### 2.2.1 General procedure for rubber functionalisation through thiol–ene reaction

The elastomer (1 g) (see Table S1† for molar quantities of –C=C– in consideration) was dissolved in a stirred nitrogen-saturated solution of toluene (5 mL) at room temperature for 24 hours. The relevant thiol and related amount of AIBN (see Table S2† for quantities) were then added to the stirring mixture, which was then left to react at 80 °C for 24 hours. The reaction was considered complete once the solution had changed from a pale yellow to a bright yellow, the final solution was then added dropwise to a vigorously stirred methanol (100 mL). The precipitated modified rubber was washed with methanol (20 mL × 3 times) and then dried at a reduced pressure for 48 hours.

#### 2.2.2 Photo-initiated thiol–ene reaction

NR (1 g, 14.7 mmol) was dissolved in a stirred nitrogen-saturated solution of dichloromethane (5 mL) at room temperature for 24 hours. TAA (1.036 mL, 14.7 mmol) and DMPA (0.188 g, 0.725 mmol) were then added. The mixture was then exposed to 62 mW cm^−2^ for one minute. The final solution was added dropwise to a vigorously stirred methanol solution (100 mL). The precipitated modified rubber was washed with methanol (20 mL × 3 times) and then dried at a reduced pressure for 48 hours.

### 2.3 Characterisation

#### 2.3.1 Dynamic differential scanning calorimetry (DSC)

Dynamic DSC was conducted to determine the glass transition temperature of the functionalised rubbers. The analysis was carried out using TA Instruments DSC25 equipment. All samples were placed in Tzero aluminium hermetic pans, and the normalized heat flow was measured for all conditions. The curing exotherms for each reactive solution were measured for 1 h at 70 °C, 80 °C and 90 °C. The thermal behaviour of the functionalised rubbers was studied by conducting heat–cool–heat experiments as follows: (1) first heating ramp from −90 °C to 200 °C at the rate of 10 °C min^−1^; (2) second cooling ramp from 200 °C to −90 °C at the rate of 5 °C min^−1^; (3) third heating ramp from −90 °C to 200 °C at the rate of 10 °C min^−1^. The *T*_g_ was calculated by evaluating the minimum point of the first derivative of the normalised heat flow.

#### 2.3.2 Average molecular weight

The number average molecular weight (*M*_n_), the weight average molecular weight (*M*_w_) and the polydispersity index (*Đ*) of NR, CR, BR and each functionalised rubber were determined by performing gel permeation chromatography (GPC) using Agilent Technologies 1260 Infinity GPC/SEC system equipped with a refractive index (RI) detector and PLgel 5 μm MIXED-C columns (300 × 7.5 mm, *M*_w_ range of 200 to 2 000 000 g mol^−1^). Calibration was carried out using polystyrene standards (*M*_w_ range from 162 g mol^−1^ to 6 570 000 g mol^−1^) from Agilent Technologies, Inc. Samples were prepared at least 48 hours prior to the analysis by dissolving approximately 10 mg of sample in 5 mL of THF. The solutions were then filtered through a 0.2 μm PTFE syringe filter and transferred into GPC vials. Each run was performed by injecting 100 μL in the columns, kept at a constant temperature of 25 °C, with a flow rate of fresh THF of 1 mL min^−1^ for 40 min.

#### 2.3.3 ^1^H and distortionless enhancement of polarisation transfer of carbons including quaternary (DEPTQ 135 ^13^C) nuclear magnetic resonance (NMR)

NMR spectroscopy was performed on a Bruker 400 MHz spectrometer at room temperature. For the analysis of the starting materials and the functionalised rubbers CDCl_3_ was used as solvent. ^1^H NMR spectra were collected performing 16 scans at 400 MHz while for ^13^C DEPTQ 135 NMR spectra 256 scans were performed at 100 MHz.

## 3 Results and discussion

**Fig. 1:**
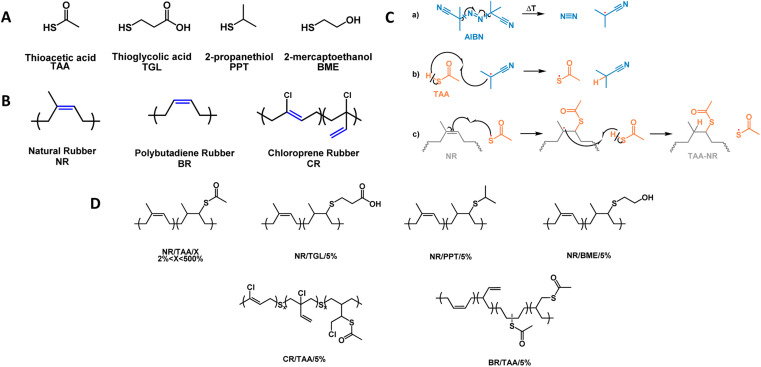
(A) Chemical structures of thiols used in this study: thioacetic acid (TAA), thioglycolic acid (TGL), 2-propanethiol (PPT), 2-mercaptoethanol (BME); (B) chemical structures of elastomers used in this study: natural rubber (NR); polybutadiene rubber (BR); chloroprene rubber (CR); (C) proposed thiol–ene reaction mechanism between TAA and NR by thermal initiation with 2,2′-azobis(2-methylpropionitrile) (AIBN), and synthesis of NR/TAA; (D) expected chemical structures of functionalised elastomers after thiol–ene reaction.

The reaction between the thiols used and unsaturated elastomers is expected to follow the anti-Markovnikov addition mechanism reported in Fig. 1D. The comparison between the GPC results obtained for NR and NR/TAA samples presented in Fig. S1A† is showing that the thiol–ene reaction with TAA had a significant impact on the measured molecular weight of corresponding reacted NRs, as both the *M*_n_ and *M*_w_ values were reduced compared to the values of non-functionalised NR (*M*_n_ = 305 000 g mol^−1^; *M*_w_ = 873 000; *Đ* = 2.8). To understand if the reduction in molecular weight was attributed to the activity of thiyl radicals, an NR sample was reacted in toluene in the presence of AIBN (molar ratio = 0.0025) but without any thiol and left at 80 °C for 24 hours. The GPC analysis reported in Fig. S1B† is suggesting that the degradation process resulting in chain reduction is a result of the activity of both AIBN and thiyl radicals, as molecular weights decreased in both cases, but more so in the presence of 50 mol% of thiol. This polymer chain degradation was also confirmed for BR and CR, as shown in Fig. S1C.† However, *Đ* was reduced in functionalised BR compared to the value obtained after NR and CR functionalisation, for which *Đ* increased. The effect of different thiols on the polymer chain degradation is shown in Fig. S1D.†

TGL functionalised NR appeared to give the lowest values of *M*_n_ and *M*_w_ compared to TAA, PPT and BME functionalised NR. This is probably due to the lower solubility of NR/TGL/5%, as the carboxylic groups have the tendency to form very strong dimers through intermolecular hydrogen bonding. Therefore, overall changes in measured molecular weights may reflect a combination of chain scission, occurring after formation of main-chain carbon radicals, and changes in molecular conformation in the solvent used for GPC (THF).

**Fig. 2:**
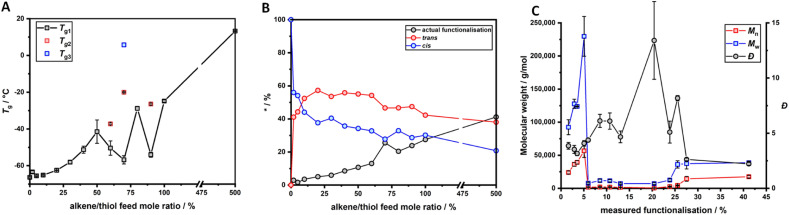
(A) Evolution of the *T*_g_ of modified NR as a function of the alkene/thiol feed mole ratio, calculated from dynamic DSC; (B) degree of *cis*/*trans* isomerism and functionalisation (in %) as a function of molar ratio of TAA used (based on ^1^H NMR); (C) evolution of *M*_n_, *M*_w_ and *Đ* of functionalised NR/TAA, as a function of the measured functionalisation level.

The *T*_g_ of the materials was determined by calculating the first derivative of the second heating ramp of each dynamic DSC experiment. This was done to maintain consistency within the different result and reduce the source of user defined errors. The *T*_g_ results are reported in Fig. 2A, displaying a marked increase as the feed molar concentration of TAA increased. For three samples, multiple *T*_g_ values are reported, presumably due to sample heterogeneity associated with some regions within the NR/TAA sample displaying higher functionalisation levels. Indeed, it is possible that more functionalised regions tended to favour further coupling, owing to the associated increased polarity, which contributes to enhancing sample heterogeneity. For the interpretation of the DSC data, the measured effective levels of functionalisation with TAA have to be considered first. ^1^H NMR data analysis was carried to quantify the amount of functionalisation. The typical ^1^H NMR spectra of NR is reported in Fig. S2,† and after functionalisation new peaks related to the thiol-functionalised NR unit are reported in Fig. S3.† The functionalisation level (A.F.%) was determined considering the integration of two distinctive peaks shown in Fig. S3A,† peaks b with *δ* = 1.53–1.61 ppm, associated to the methyl hydrogens of the NR units, and peak j with *δ* = 2.25 ppm, associated to the methyl hydrogens of the thiolated NR units, using eqn (1):1
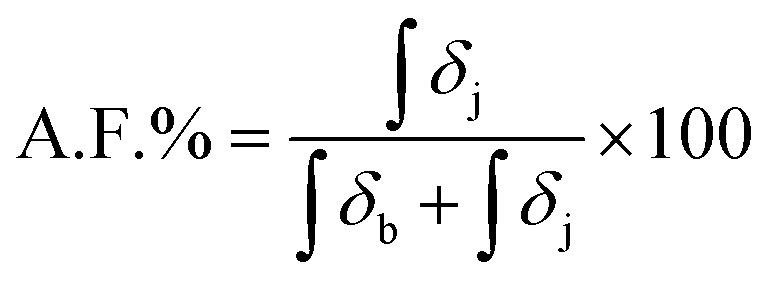


Similarly, the functionalisation degree was calculated for NR/TGL/5%, NR/PPT/5% and NR/BME/5%, based on comparable peak attribution reported in Fig. S3B–D,† and eqn (1) for quantification. In addition, peaks b in Fig. S3,† attributed to the hydrogens of the –CH_3_ group of the non-functionalised NR, were found to split into two peaks after functionalisation, suggesting the occurrence of isomerism from *cis* to *trans* configuration of the double bond. To quantify the extent of *cis*/*trans* isomerism, the integration of the two b peaks was analysed using eqn (2), where the isomer in *cis* configuration was attributed *δ* = 1.61 ppm, while to the isomer in *trans* configuration was assigned *δ* = 1.53 ppm:2
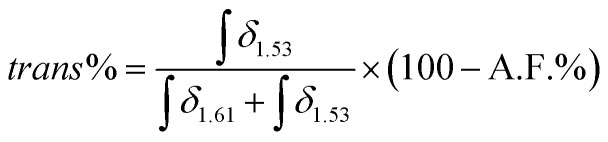


**Fig. 3:**
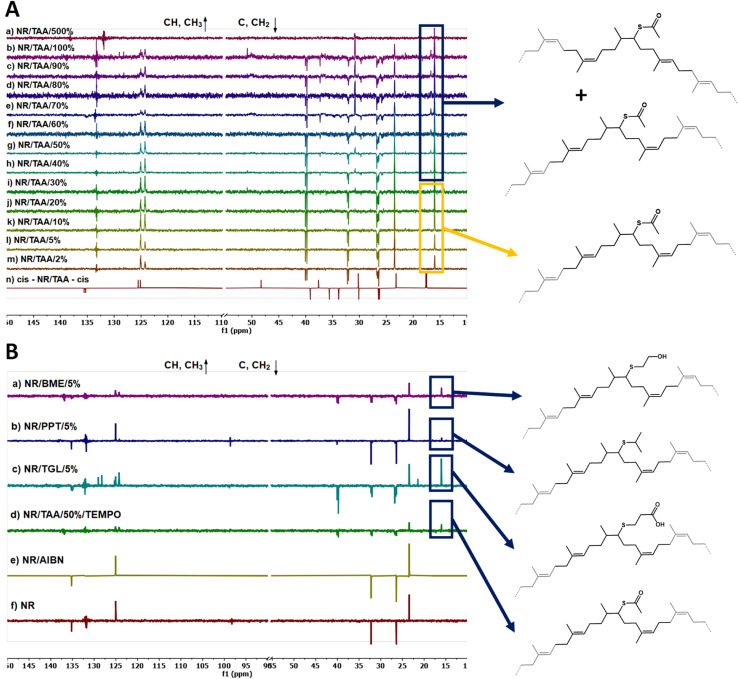
(A) ^13^C NMR (100 MHz in CDCl_3_) spectra of NR/TAA/*X* at different thiol feed ratios; (B) ^13^C NMR (100 MHz in CDCl_3_) spectra of (a) NR/BME/5%, (b) NR/PPT/5%, (c) NR/TGL/5%, (d) NR/TAA/50%/TEMPO, (d) NR/AIBN and (e) NR.

The calculated values of actual functionalisation degree and *cis*/*trans* isomerism are reported in Table S3.†^13^C NMR characterisation was used to analyse and determine the position of these *cis* and *trans* repeats relative to TAA-functionalised NR repeats. For this purpose, the prediction tools provided by MestRenova software were used. The acquired ^13^C NMR spectra for NR/TAA/*X* were particularly complex, not only due to the *cis* and *trans* isomerism, as functionalisation with TAA clearly perturbed the electronic density of backbone carbons and provided a range of chemical environments for the carbons of neighbouring repeats. To interpret the acquired DEPTQ ^13^C NMR spectra of NR/TAA/*X*, the predicted impact of various sequences of repeat units possible were determined on chemical shifts. Firstly it was established that different sequences of non-functionalised polyisoprene units do not affect the chemical shifts characterising the central unit (see Fig. S4† for structures of δ *trans*-TRANS-*trans* = δ *cis*-TRANS-*trans* = δ *cis*-TRANS-*cis* = δ *trans*-TRANS-*cis* and Fig. S5† for ^13^C NMR prediction; Fig. S6† for structures of δ *cis*-CIS-*cis* = δ *cis*-CIS-*trans* = δ *trans*-CIS-*trans* = δ *trans*-CIS-*cis* and Fig. S7† for DEPTQ ^13^C NMR prediction) while different surroundings of TAA-functionalised NR units (abbreviated to -TAA- when between two different isomers of NR, for example *cis* NR unit – functionalised NR – *cis* NR unit = *cis*-TAA-*cis*) do result in different ^13^C NMR patterns, as shown in the predictions reported in Fig. S10† for *cis*-TAA-*cis*, Fig. S13† for *cis*-TAA-*trans*, Fig. S16† for *trans*-TAA-*trans* and Fig. S19† for *trans*-TAA-*cis*. The acquired ^13^C NMR spectra for NR/TAA/*X* reported in Fig. 3A show similarities with the predicted spectrum of *trans*-TAA-*cis* from NR/TAA/2% to NR/TAA/30%, within the range of 15 and 17.5 ppm suggesting the presence of the *trans*-TAA-*cis* form, while from NR/TAA/40% to NR/TAA/100% the *cis*-TAA-*trans* form also seems to be present. As at a lower degree of functionalisation with TAA the *trans*-TAA-*cis* configuration is obtained, it was expected that thiol–ene functionalisation with TGL, PPT and BME would result in a similar distribution of repeat unit sequences. Fig. 3B, reporting the ^13^C NMR spectra of NR/TGL/5%, NR/PPT/5% and NR/BME/5% confirmed this hypothesis. Samples treated with AIBN only (no thiol) did not show any *cis*/*trans* isomerism, confirmed by the ^1^H NMR spectra reported in Fig. S20.†

**Fig. 4:**
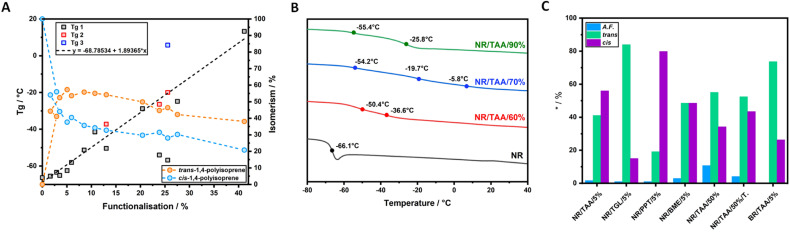
(A) Linear correlation between the measured functionalisation determined through ^1^H NMR and evolving *T*_g_ of modified NR gathered from dynamic DSC analysis. On the right *Y*-axis the *cis*/*trans* isomerism is reported (B) second heating ramp of dynamic DSC of NR/TAA/60%, NR/TAA/70% and NR/TAA/90% compared to NR; (C) degree of *cis*/*trans* isomerism and functionalisation (A.F. in %) comparison between NR/TAA/5%, NR/TGL/5%, NR/PPT/5% and NR/BME/5%; comparison between NR/TAA/50% and NR/TAA/50%/TEMPO; *cis*% and *trans*% values of BR/TAA/5%.

Having quantified the functionalisation levels of NRs, these results are correlated with the changes in the thermal properties observed by DSC. According to the dynamic DSC data analysis reported in Fig. 2A, a clear correlation between the degree of functionalisation achieved in each sample and the shift in *T*_g_ when compared to pure NR was observed. To confirm this, the *T*_g_ of each sample was plotted against the measured functionalisation degree calculated from ^1^H NMR spectra. As shown in Fig. 4A, the *T*_g_ values linearly correlated with the measured degree of thiol coupling. A significant amount of *cis*/*trans* isomerism appeared to take place even at a very low degree of functionalisation, reaching a ratio close to 50/50 with higher concentration of *cis* isomer, already at 2% of functionalisation. Starting from sample NR/TAA/10%, where the actual degree of functionalisation achieved is 3.5%, the *trans* isomer has become more dominant (Fig. 4A). This likely reflects the increased concentration of thiyl radicals associated with higher thiol and AIBN feed ratios, and the resulting increased rates of formation of carbon radicals underpinning the isomerism. Since the NR sample treated with AIBN-only did not show any *cis*/*trans* isomerism, the equilibrium seems to only require the presence of thiyl radicals in this context. This is in good agreement with the proposal that the thiyl addition is reversible and result in the effective isomerism.^57,58^ In contrast, the degradation mechanism suggested by the GPC results, with significantly reduced *M*_n_, *M*_w_ and increased dispersity, was not found to require the presence of thiols and thiyl species. This implies that low concentrations of carbon radicals formed from direct reaction of AIBN decomposition products destabilise sufficiently C–C bonds of the polymer backbone, but that the lack of reversibility of their formation cannot underpin isomerisation.

With respect to the functionalised samples displaying high heterogeneity, it could be possible to interpolate the actual degree of functionalisation of the phase exhibiting the anomalous *T*_g_ from the linear regression determined by considering only the samples displaying a single *T*_g_. In the set of experiments discussed in this research, three samples showed the tendency to have multiple *T*_g_ values: NR/TAA/60%, NR/TAA/70% and NR/TAA/90%. The tendency to have multiple *T*_g_ was maintained in the second heat cycle of dynamic DSC analysis, as reported in Fig. 4B (first derivative calculation reported in Fig. S21†), suggesting that the heterogeneity could be due to a phase separation due to different levels of functionalisation leading to different polarities within the sample.

*Cis*/*trans* isomerism is also observed in TGL, PPT and BME treated samples, but to a different extent. As reported in Fig. 4C, when NR is treated with different thiols at the same concentration, this would not result in the same *cis*/*trans* isomerism. In NR/PPT/5% the *cis*/*trans* equilibrium is shifted towards the *cis* isomer, in NR/TGL/5% the *trans* isomer is dominant, while in NR/BME/5% a 50/50 ratio is achieved. The strong predominance of *cis* isomer in NR/PPT/5% could be due to the steric hindrance of the PPT derived thiyl radical. In addition, the electron donating character of PPT may stabilise the resulting carbon radical intermediates (after addition to olefins), limiting the rate of scission and isomerisation. Therefore, the combination of steric effects and stabilisation of the electron cloud associated with carbon radical intermediate may underpin the differences in isomerisation rates observed.

The ^1^H NMR spectrum of BR/TAA/5% does not indicate any measurable functionalisation degree, but the exposure to thiol–ene reaction conditions yielded significant *cis*/*trans* isomerism (Fig. 4C and Table S3†). This behaviour is proposed to result from the lack of tertiary carbon in the backbone of BR, reducing the stability of intermediate and therefore effective thiol–ene coupling, yet enabling sufficient level of thiyl addition to sustain isomerism. Indeed, a comparison between the ^1^H NMR spectrum of BR/TAA/5% and the ^1^H NMR spectrum of BR is reported in Fig. S22A.†^1^H NMR of BR is reporting the presence of a small percentage of 1,2-units, highlighted in blue. After reaction with TAA, peaks related to the 1,4-units appear, highlighted in yellow. New broad peaks also appear in the region between 1.2 ppm and 1.8 ppm and around 3.5 ppm, proposed to correspond to the protons of the functionalised unit highlighted in red. The reaction between CR and TAA did not make any visible changes in the ^13^C NMR spectra analysed. However, the ^1^H NMR spectra analysis did report some changes, although none were indicative of the thiol–ene reaction under investigation. According to the literature,^72^ it is possible to assign different chemical shifts to different series of configuration. The structures under examination are reported in Fig. S22B,† along with a comparison between CR/TAA/5% and CR ^1^H NMR spectra. The spectrum of CR/TAA/5% is only showing a cleaner CR. The spectrum of CR does not report signals relative to the *cis*-1,4 units, while peaks relative to the hydrogens in 1,2 units are present, even if these are at a small concentration when compared to the *trans*-1,4 units.

In turn, changes in *T*_g_ of BR and CR after functionalisation with TAA were also observed, however the dynamic DSC thermogram of BR and BR/TAA/5%, reported in Fig. S23A† were very different from the typical dynamic DSC thermogram obtained from NR and the functionalised NR products, while CR and CR/TAA/5%, reported in Fig. S23B† showed similar behaviour to NR and thiol functionalised NR compounds. For BR, peaks relative to crystal melting, *T*_m_, were observed, but *T*_g_ was not detected under these conditions, as it is known to be below the temperature range tested here at around −104 °C.^73,74^ BR/TAA/5% showed different behaviour: an exothermic peak around −50 °C, followed by a broad endothermic peak. This could be due to the degree of isomerism achieved. CR on the other hand, showed a very small shift of *T*_g_ from −41.7 °C prior to treatment to −39.9 °C after treatment.

## 4 Conclusions

**Fig. 5:**
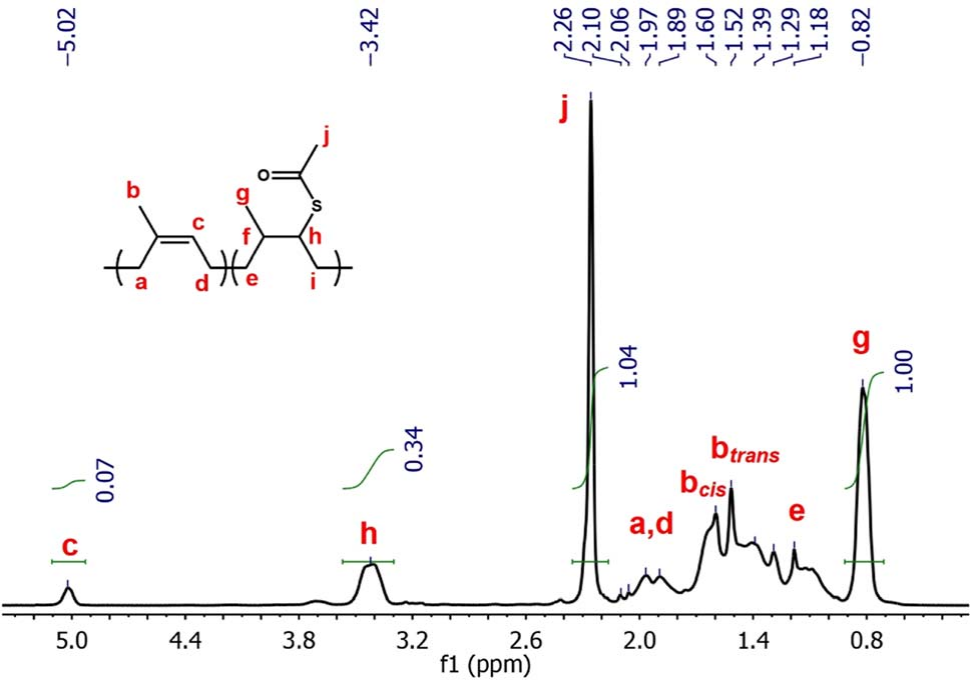
^1^H NMR 400 MHz of NR/TAA in CDCl_3_ obtained through photoinitiation in dichloromethane with DMPA at 62 mW cm^−2^ for 1 minute.

In summary, the thiol–ene reaction is an effective tool that can be exploited for the development of novel functionalisation and cross-linking methods for main chain olefinic elastomers, allowing progress into recyclability and longer application lifetime. It must be noted that the thiol–ene reaction between the different elastomers and thiols was carried at 80 °C for 24 hours. Although the thermally initiated thiol–ene reaction is considered to be a clean and efficient reaction, the involved free radicals would undoubtedly participate in side reactions, such as chain scission of the polymer chains, explaining the observed drop in molecular weight from the GPC analysis. It could be argued that shorter reaction time would diminish this degradative effect, however it may lead to even lower measured functionalisation values, unless a photoinitiated approach is adopted. In Fig. 5 the spectra of the DMPA initiated thiol–ene reaction equivalent to NR/TAA/100% is reported. The resulting product had a much higher A.F. compared to the thermally initiated counterpart (82.9% *vs.* 27.5%) and it was calculated the following eqn (3) where peak c and h were compared:3
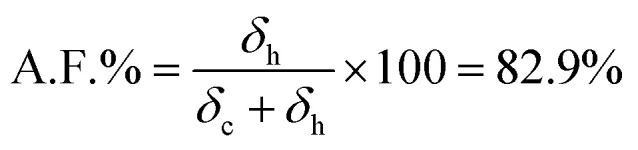


The calculation of the *cis*/*trans* isomerism however is not reported as the peaks related to b_*cis*_ and b_*trans*_ are overlapping with the multiplets from e and i; however, the ratio between *cis* and *trans* isomers seems to be shifted towards the *trans* isomers. In the thiol–ene reaction investigation carried out on NR with TAA, TGL, PPT and BME, thiyl-induced *cis*/*trans* isomerism was observed in all compounds, with different extents depending on the thiol used. The *T*_g_ of the resulting functionalised elastomers proportionally increased with the degree of functionalisation. At higher degrees of functionalisation, a phase segregation phenomenon was proposed to occur, enhancing heterogeneity of samples and resulting in multiple *T*_g_ values (presumably associated with each phase). As *M*_n_ decreases of several orders of magnitude 10^5^ to 10^2^ with increasing degree of functionalisation, the *T*_g_ of the resulting functionalised elastomers should decrease according to the Flory–Fox^75,76^ equation, stating that the free volume should increase with increasing number of chain-ends in a given volume.^77^ Given the results obtained from thiol–ene functionalisation where the *T*_g_ increases with increasing degree of functionalisation, it is clear that *T*_g_ is influenced by counterbalancing effect, where reduction of *M*_n_ should push towards increasing free volume between chains, while increasing functionalisation has the opposite and dominant effect. ^13^C NMR analysis results show a preferred configuration arrangement between functionalised and non-functionalised units at lower functionalisation degree, the *trans*-TAA-*cis* configuration, while at higher functionalisation degree a secondary configuration starts forming, the *cis*-TAA-*trans* configuration. The *cis*/*trans* isomerism was confirmed to be specifically associated with exposure to thiyl radicals, as a result of reversible addition of thiyl species to main chain olefins. The dynamic DSC analysis showed a change in thermal behaviour after reaction with TAA, but further analysis is required to understand the abnormal peaks observed between −70 °C and 20 °C, as most probably they are related to significant changes in crystallinity as *trans* units are forming. CR did not react with TAA to a sufficient extent to use NMR analysis to assess the functionalisation degree. Therefore, in addition to potential for functionalisation, thiol–ene conditioning may be an interesting and relatively simple route to isomerisation of rubbers, although chain degradation will have to be limited for application of such systems.

## Author contributions

Anureet Kaur: investigation, methodology, data curation, writing, review & editing. Julien E. Gautrot: methodology, conceptualisation, supervision, review & editing. Keizo Akutagawa: methodology, conceptualisation, supervision, review & editing. Douglas Watson: conceptualisation, supervision. Alan Bickley: conceptualisation, supervision. James J. C. Busfield: methodology, supervision, writing, review & editing.

## Conflicts of interest

There are no conflicts to declare.

## Supplementary Material

RA-013-D3RA04157C-s001
