# Supplementary material for: Thiyl radical induced cis/trans isomerism in double bond containing elastomers
Source: RSC Adv. 2023 Aug 10;13(34):23967–75. doi: 10.1039/d3ra04157c (PMC10413178; doi:10.1039/d3ra04157c)
Supplement: RA-013-D3RA04157C-s001 [file RA-013-D3RA04157C-s001.pdf]

Electronic Supplementary Material (ESI) for RSC Advances.
This journal is © The Royal Society of Chemistry 2023

Supplementary Material

Thiyl Radical Induced Cis/Trans Isomerism in Double Bond Containing Elastomers†

Anureet Kaur[a], Julien E. Gautrot[a], Keizo Akutagawa[a], Douglas Watson[b], Alan Bickley[b] and James J. C. Busfield*[a]

[a] *Queen Mary University of London, London, United Kingdom.*
E-mail: anureet.kaur@qmul.ac.uk, j.gautrot@qmul.ac.uk, g.cavalli@qmul.ac.uk, k.akutagawa@qmul.ac.uk, j.busfield@qmul.ac.uk.
[b] *Weir Advanced Research Centre, Glasgow, United Kingdom.*
E-mail: douglas.watson@mail.weir, alan.bickley@mail.weir;.

Table S1 Double bond content calculation in NR, BR and CR.

	Weight	Molar Mass	–C=C–
	**g**	**g/mol**	**mol**
**NR**	**1**	68.119	0.0147
**CR**	**1 (0.994 g of CR)**	88.534	0.0112
**BR**	**1**	54.092	0.0185

Table S2 Quantities of each material involved in the thiol-ene based functionalisations. (M.R. - molar ratio)

	–C=C–	Thiol	TAA	TGL	PPT	BME	AIBN		
		**M.R.**	**mL**	**mL**	**mL**	**mL**	**M.R.**	**mol**	**g**
NR (1 g)	**0.0147**	0.02	0.021				0.001	1.47E-05	0.0024
		0.05	0.052	0.051	0.068	0.052	0.0025	3.68E-05	0.0060
		0.1	0.104				0.005	7.35E-05	0.0121
		0.2	0.207				0.01	1.47E-04	0.0241
		0.3	0.311				0.015	2.21E-04	0.0362
		0.4	0.414				0.02	2.94E-04	0.0483
		0.5	0.518				0.025	3.68E-04	0.0603
		0.6	0.622				0.03	4.41E-04	0.0724
		0.7	0.725				0.035	5.15E-04	0.0845
		0.8	0.829				0.04	5.88E-04	0.0966
		0.9	0.932				0.045	6.62E-04	0.1086
		1	1.036				0.05	7.35E-04	0.1207
		5	5.180	5.106	6.827	5.155	0.25	3.68E-03	0.6035
CR (1 g)	**0.0112**	0.05	0.039				0.0025	2.80E-05	0.0046
BR (1 g)	**0.0185**	0.05	0.065				0.0025	4.63E-05	0.0076

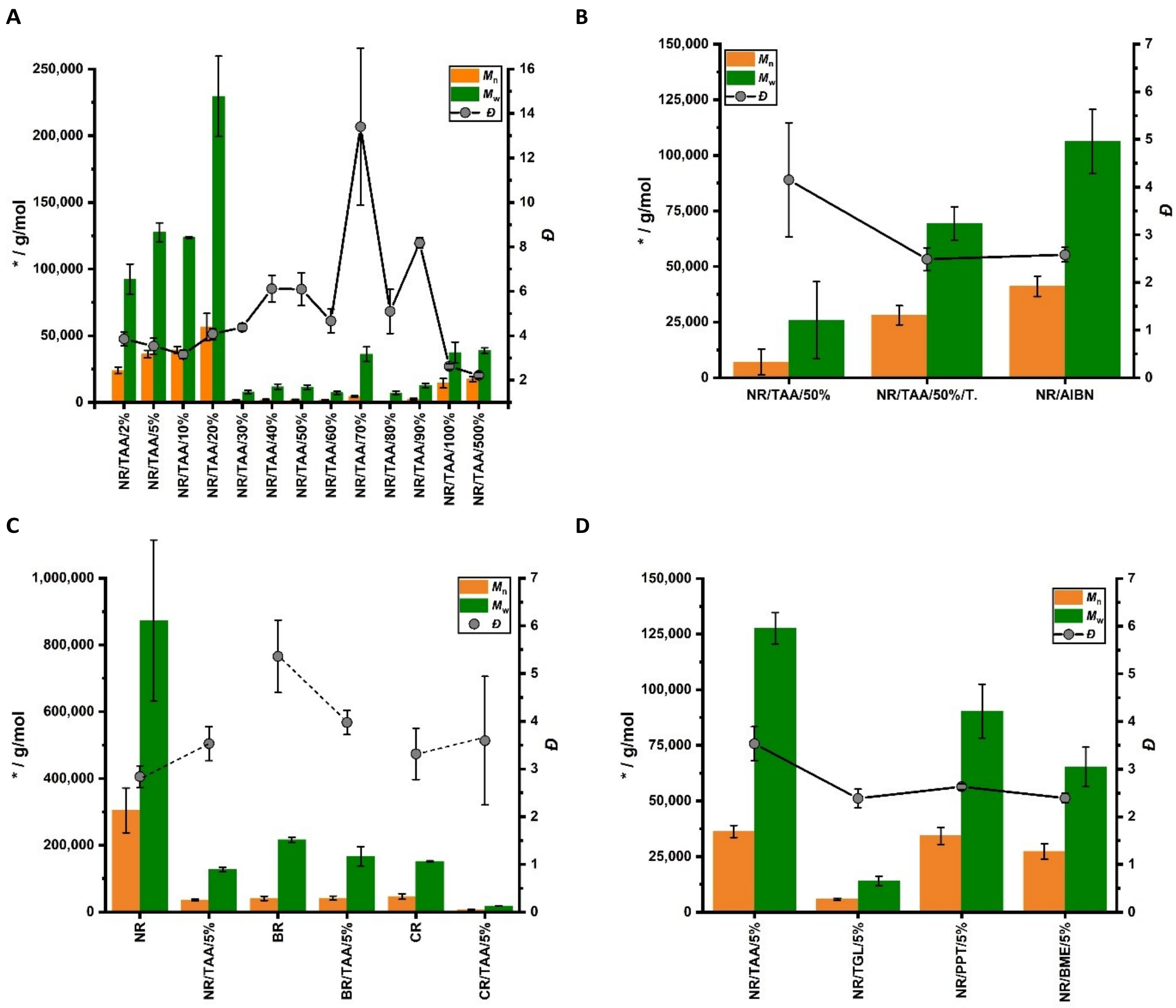

Figure S1 (**A**) Evolution of M_n, M_w and $Đ$ of modified NR as a function of the alkene/thiol feed mole ratio; (**B**) GPC analysis comparison between NR, NR/TAA/50% treated with TEMPO and NR treated with AIBN only; (**C**) GPC results comparison between elastomers and TAA- functionalised elastomers with different thiols used alkene/thiol feed mole ratio of 5%; (**D**) GPC analysis comparison between different thiol functionalised NR.

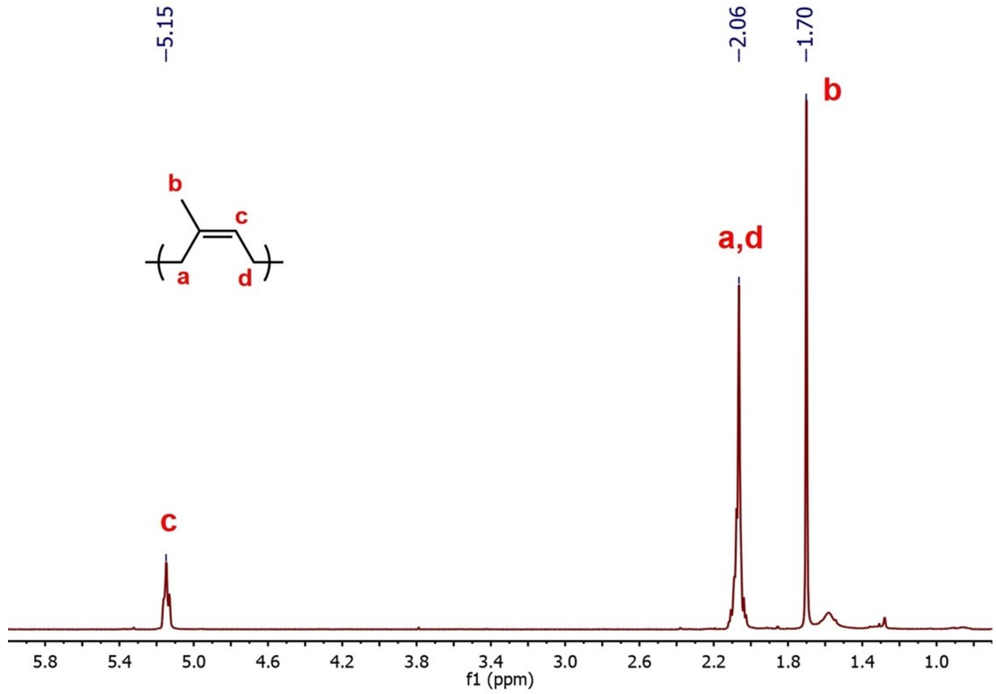

Figure S2 Typical ^{1}H NMR 400 MHz of NR in $CDCl_3$

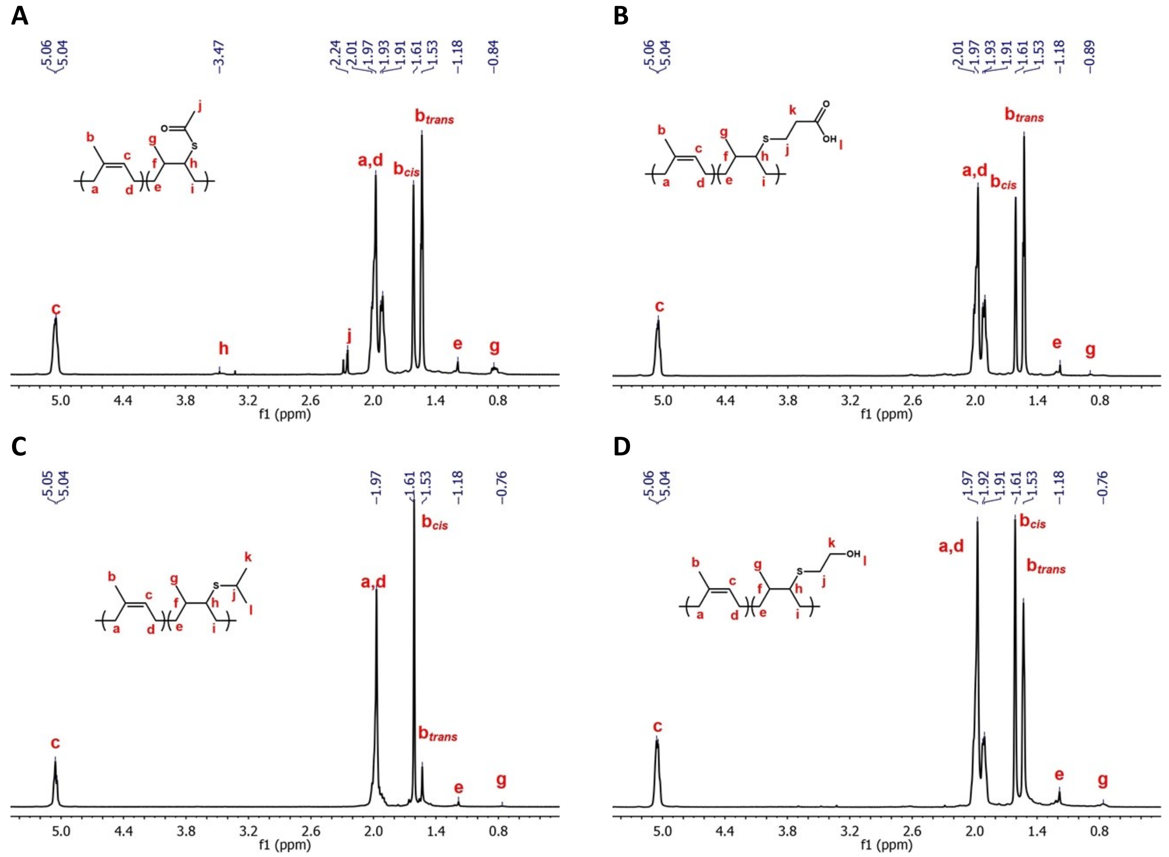

Figure S3 (**A**) Typical ^{1}H NMR 400 MHz of NR/TAA in $CDCl_3$; (**B**) ^{1}H NMR 400 MHz of NR/TGL/5% in $CDCl_3$; (**C**) ^{1}H NMR 400 MHz of NR/PPT/5% in $CDCl_3$; (**D**) ^{1}H NMR 400 MHz of NR/BME/5% in $CDCl_3$

Table S3 Actual functionalisation values, percentage of trans and cis isomerism in each compound; NR/TAA/2% is showing a higher degree of functionalisation than expected, probably due to overlapping peaks in collected ^{1}H NMR spectra.

	A.F. / %	trans / %	cis / %
NR	-	-	~100
NR/TAA/2%	2.91	41.10	55.99
NR/TAA/5%	1.64	44.26	54.10
NR/TAA/10%	3.54	52.41	44.05
NR/TAA/20%	5.06	57.28	37.66
NR/TAA/30%	5.96	53.61	40.43
NR/TAA/40%	8.54	55.79	35.67
NR/TAA/50%	10.71	55.06	34.23
NR/TAA/60%	13.04	54.20	32.76
NR/TAA/70%	25.56	46.40	28.04
NR/TAA/80%	20.42	49.87	29.71
NR/TAA/90%	23.86	44.67	31.47
NR/TAA/100%	27.53	42.27	30.19
NR/TAA/500%	41.18	38.04	20.78
NR/TAA/50%/T.	4.15	52.40	43.45
NR/TGL/5%	0.99	83.98	15.03
NR/PPT/5%	0.99	19.14	79.87
NR/BME/5%	2.8	48.54	48.54
BR	-	-	~98
BR/TAA/5%	-	73.68	26.32
CR	-	~90	-
CR/TAA/5%	-	~90	-

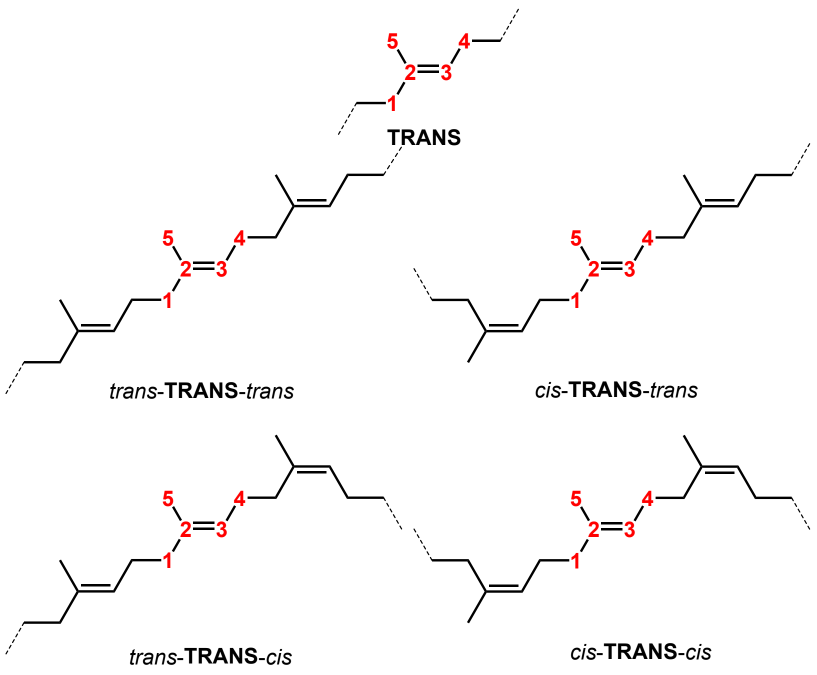

Figure S4 Structures of trans-TRANS-trans, cis-TRANS-trans, trans-TRANS-cis and cis-TRANS-cis.

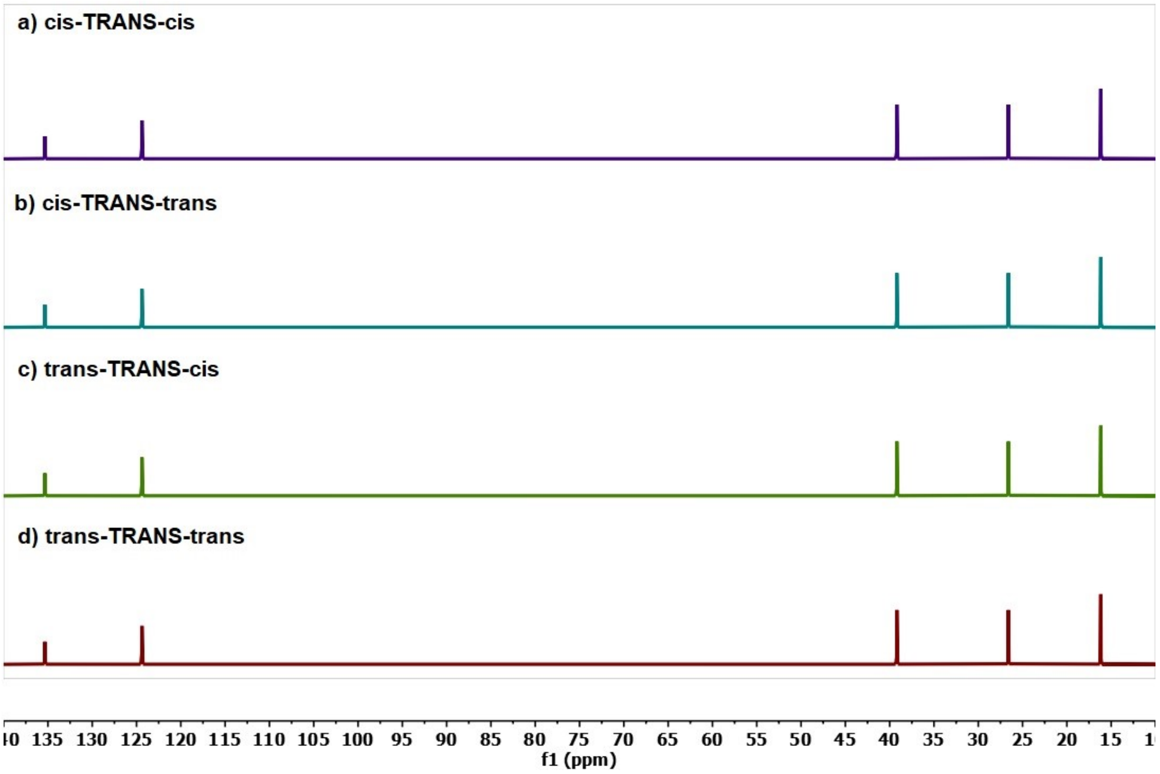

Figure S5 ^{13}C NMR predictions of **a)** cis-TRANS-cis, **b)** cis-TRANS-trans, **c)** trans-TRANS-cis and **d)** trans-TRANS-trans.

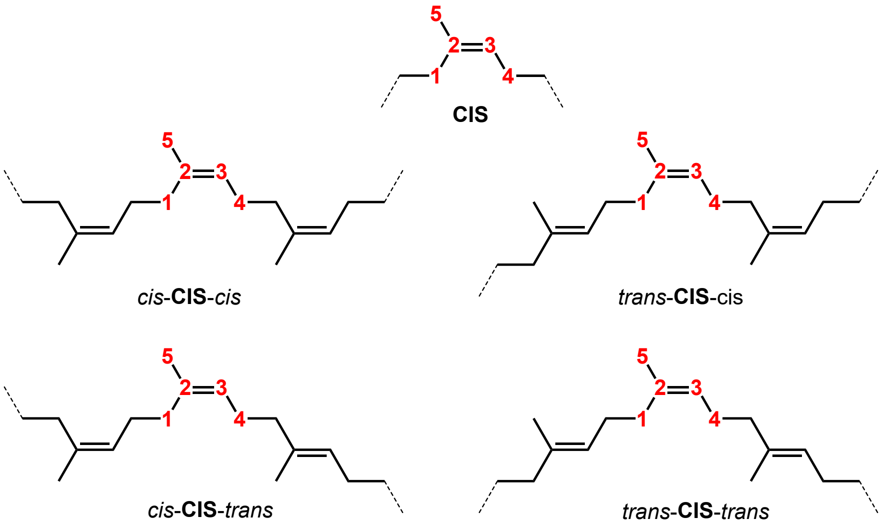

Figure S6 Structures of cis-CIS-cis, cis-CIS-trans, trans-CIS-trans and trans-CIS-cis.

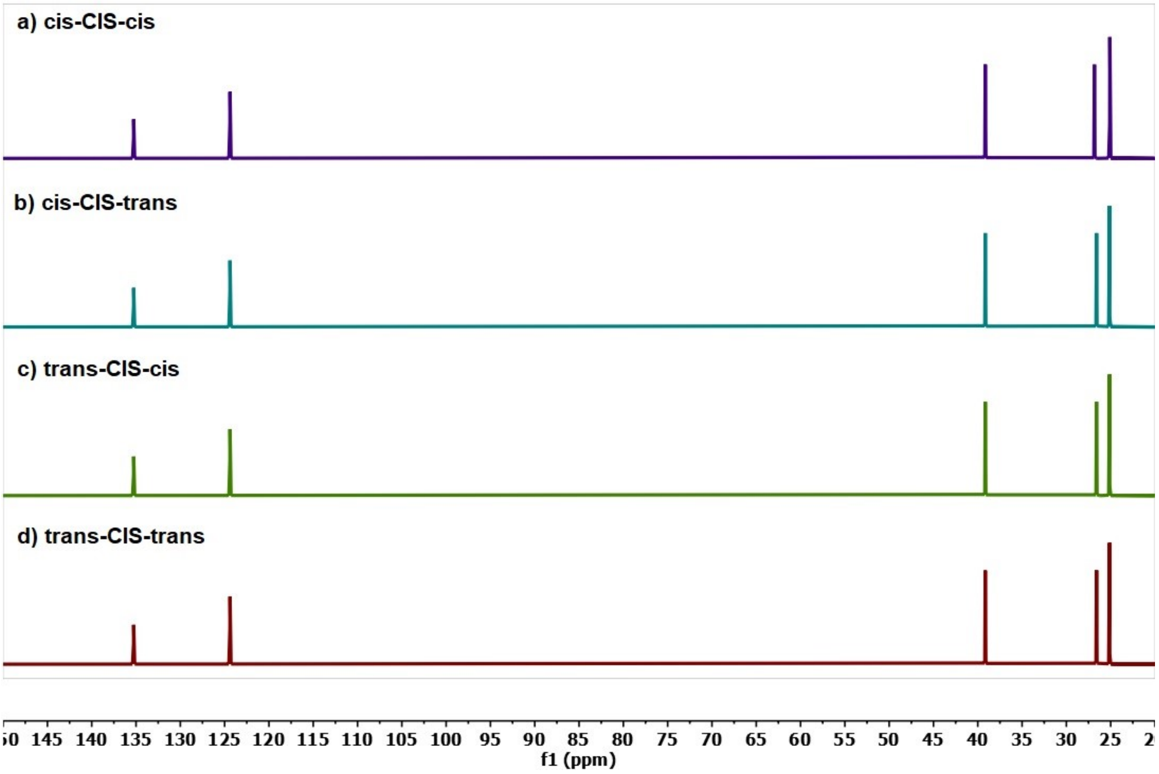

Figure S7 ^{13}C NMR predictions of **a)** cis-CIS-cis, **b)** cis-CIS-trans, **c)** trans-CIS-cis and **d)** trans-CIS-trans.

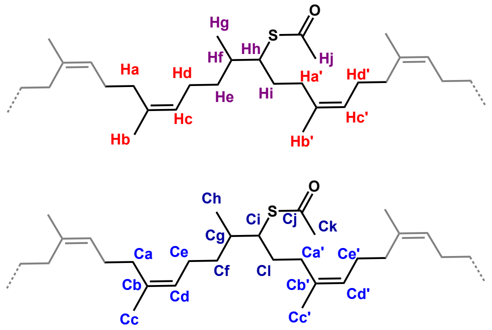

Figure S8 Chemical structure of NR/TAA in cis-TAA-cis configuration for ^{1}H NMR and ^{13}C NMR interpretation.

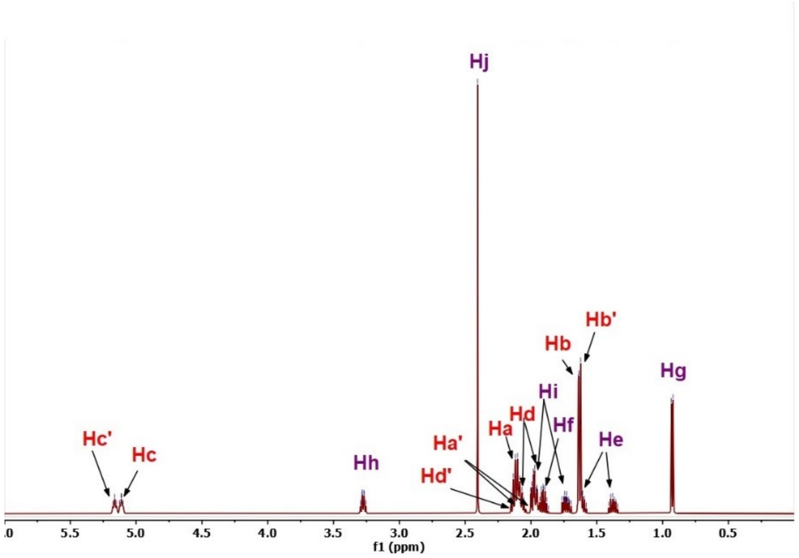

Figure S9 ^{1}H NMR prediction for NR/TAA in cis-TAA-cis configuration.

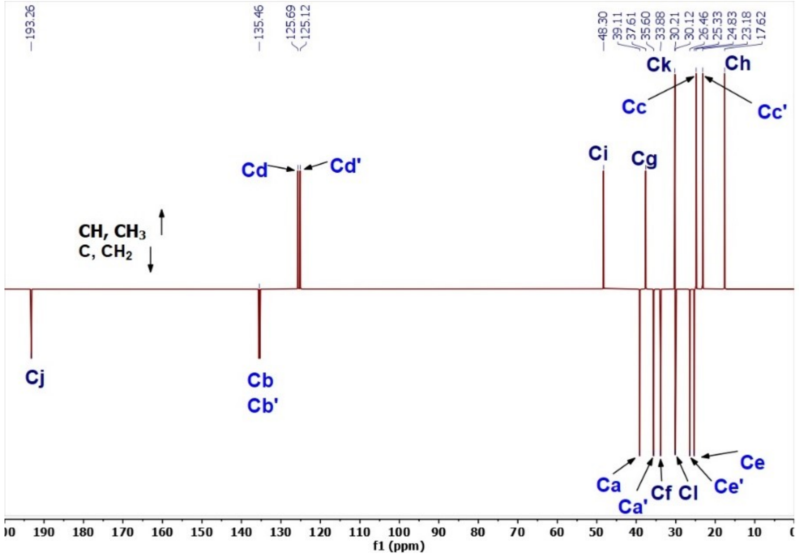

Figure S10 ^{13}C NMR prediction for NR/TAA in cis-TAA-cis configuration.

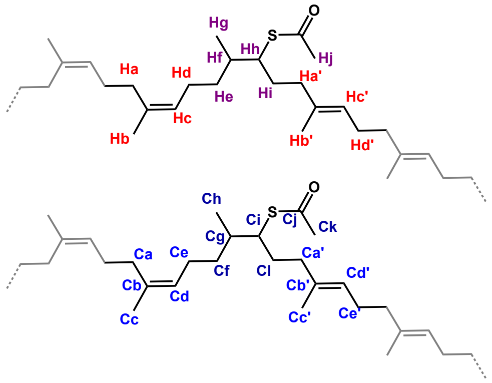

Figure S11 Chemical structure of NR/TAA in cis-TAA-trans configuration for ^{1}H NMR and ^{13}C NMR interpretation.

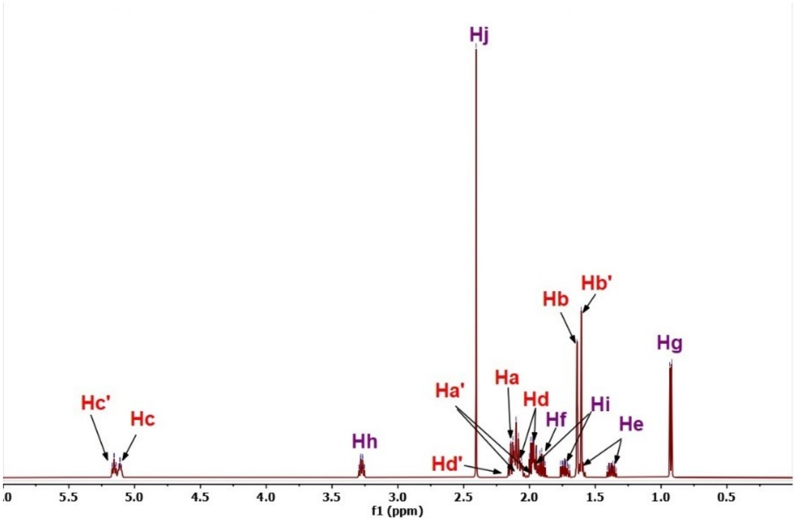

Figure S12 ^{1}H NMR prediction for NR/TAA in cis-TAA-trans configuration.

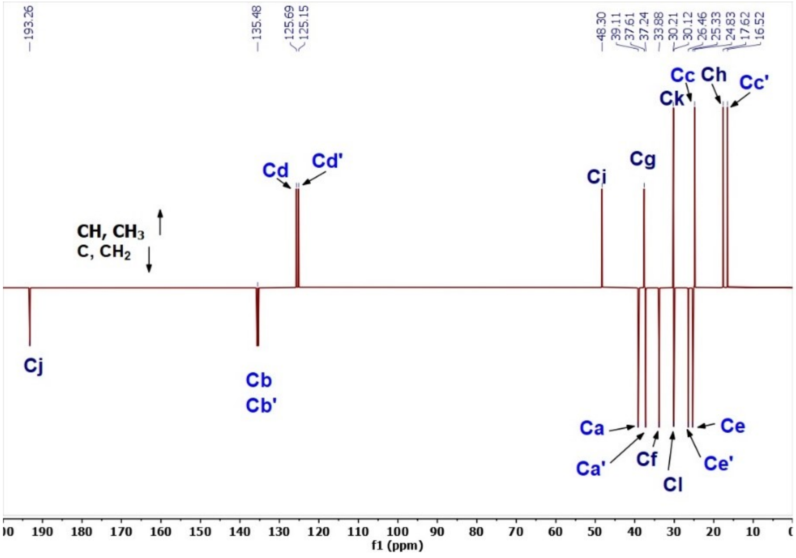

Figure S13 ^{13}C NMR prediction for NR/TAA in cis-TAA-trans configuration.

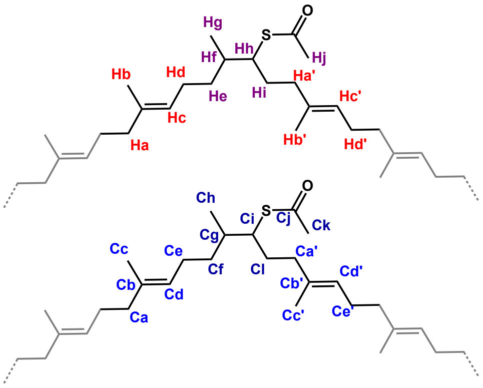

Figure S14 Chemical structure of NR/TAA in trans-TAA-trans configuration for ^{1}H NMR and ^{13}C NMR interpretation.

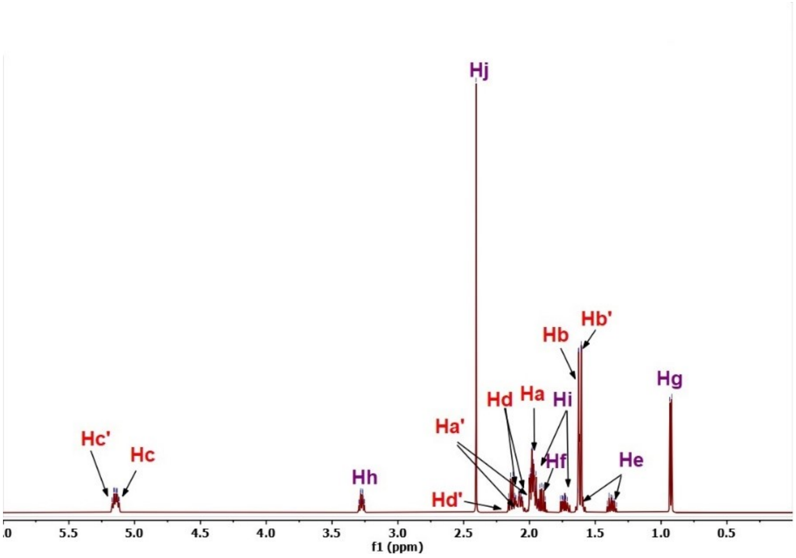

Figure S15 ^{1}H NMR prediction for NR/TAA in trans-TAA-trans configuration.

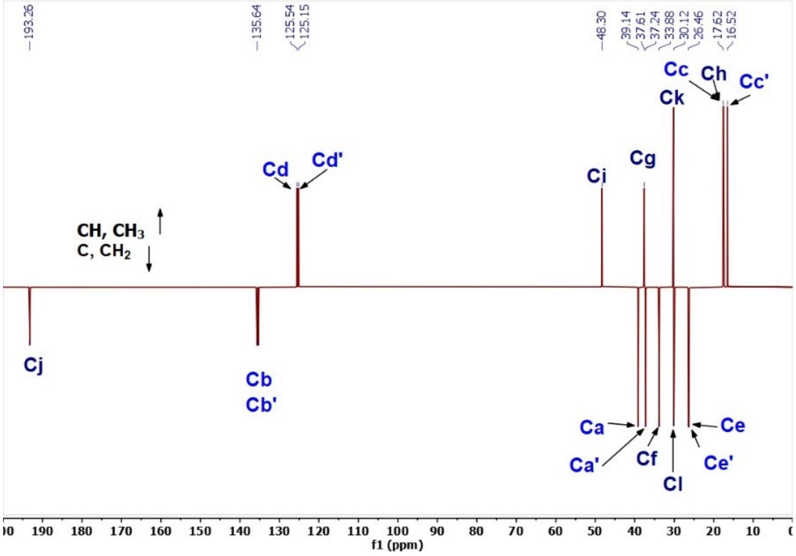

Figure S16 ^{13}C NMR prediction for NR/TAA in trans-TAA-trans configuration.

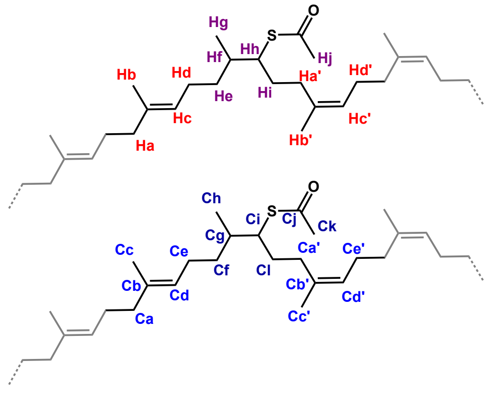

Figure S17 Chemical structure of NR/TAA in trans-TAA-cis configuration for ^{1}H NMR and ^{13}C NMR interpretation.

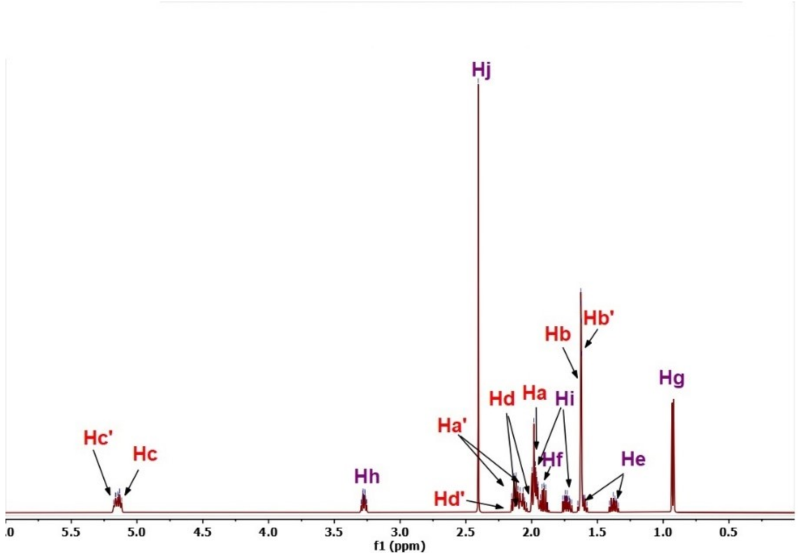

Figure S18 ^{1}H NMR prediction for NR/TAA in trans-TAA-cis configuration.

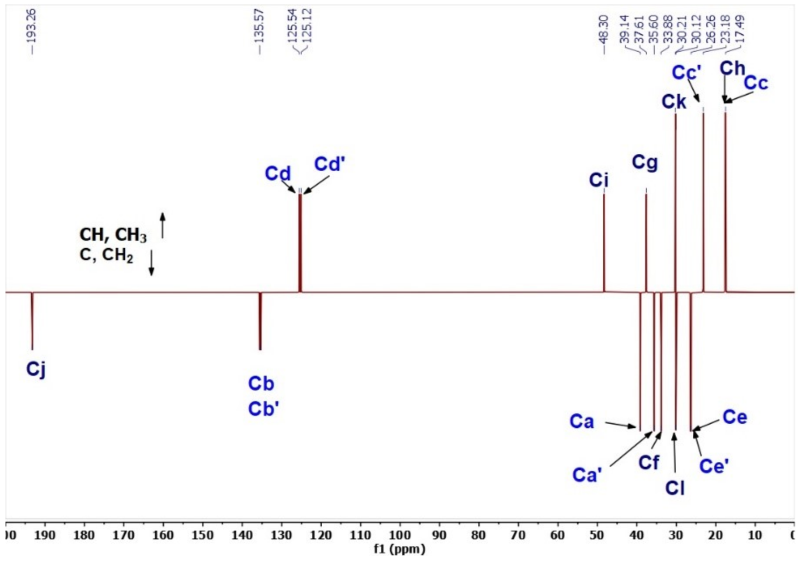

Figure S19 ^{13}C NMR prediction for NR/TAA in trans-TAA-cis configuration.

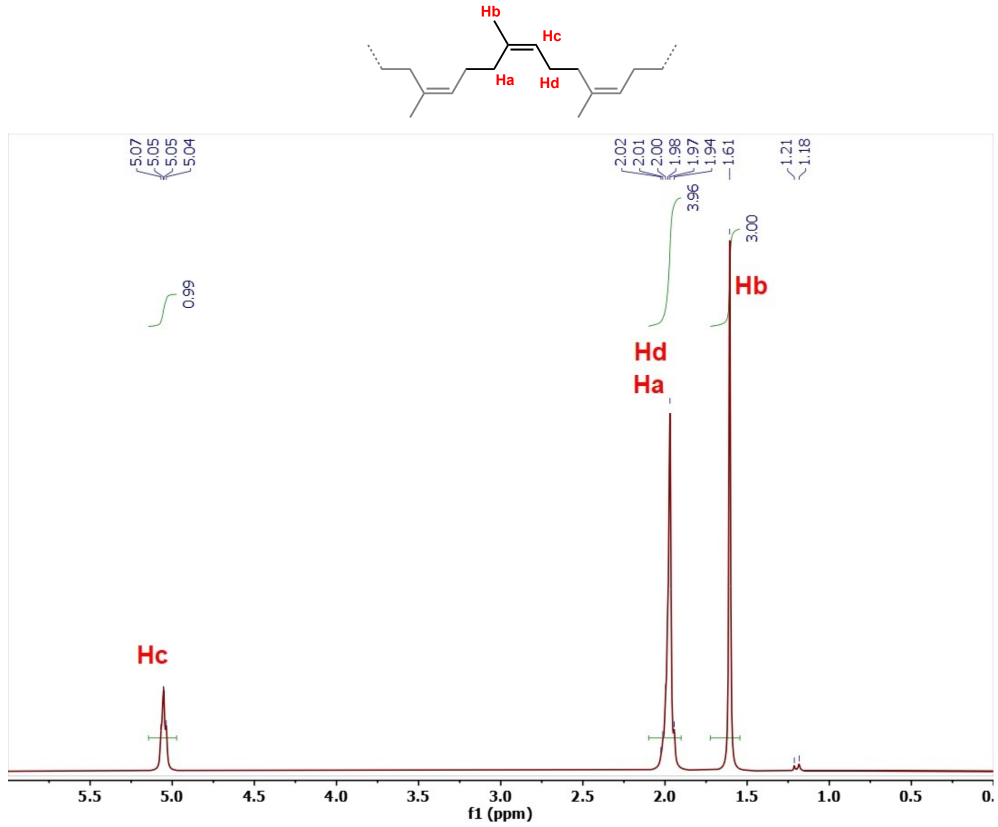

Figure S20 ^{1}H NMR 400 MHz in $CDCl_3$ of NR/AIBN.

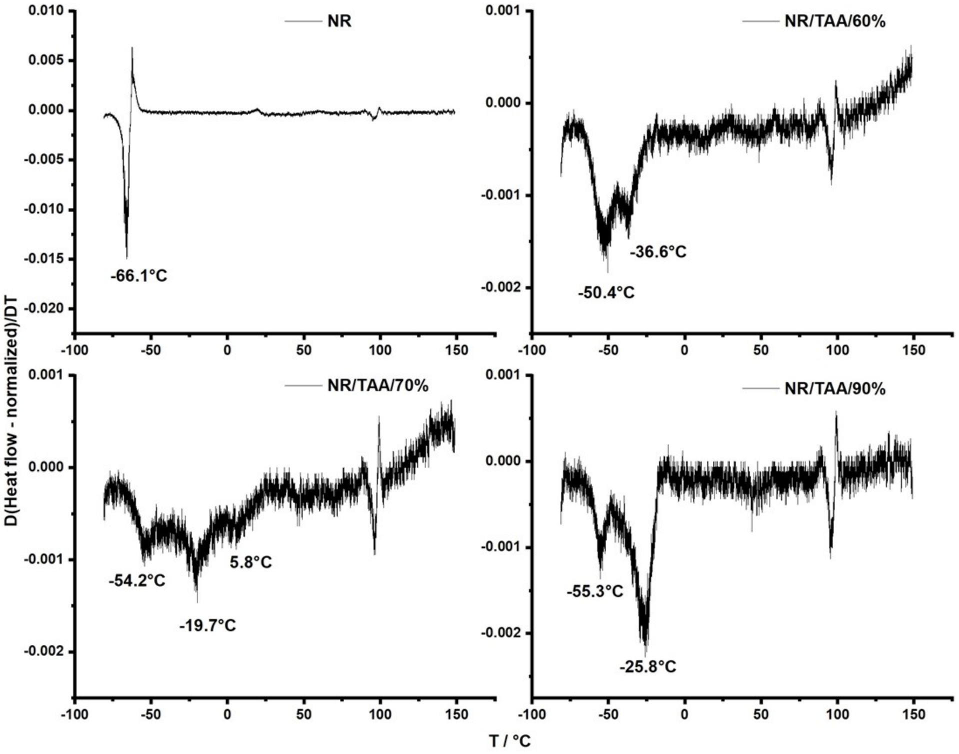

Figure S21 First derivative calculation of second heating ramp of dynamic DSC to determine T_g of NR, NR/TAA/60%, NR/TAA/70% and NR/TAA/90%;

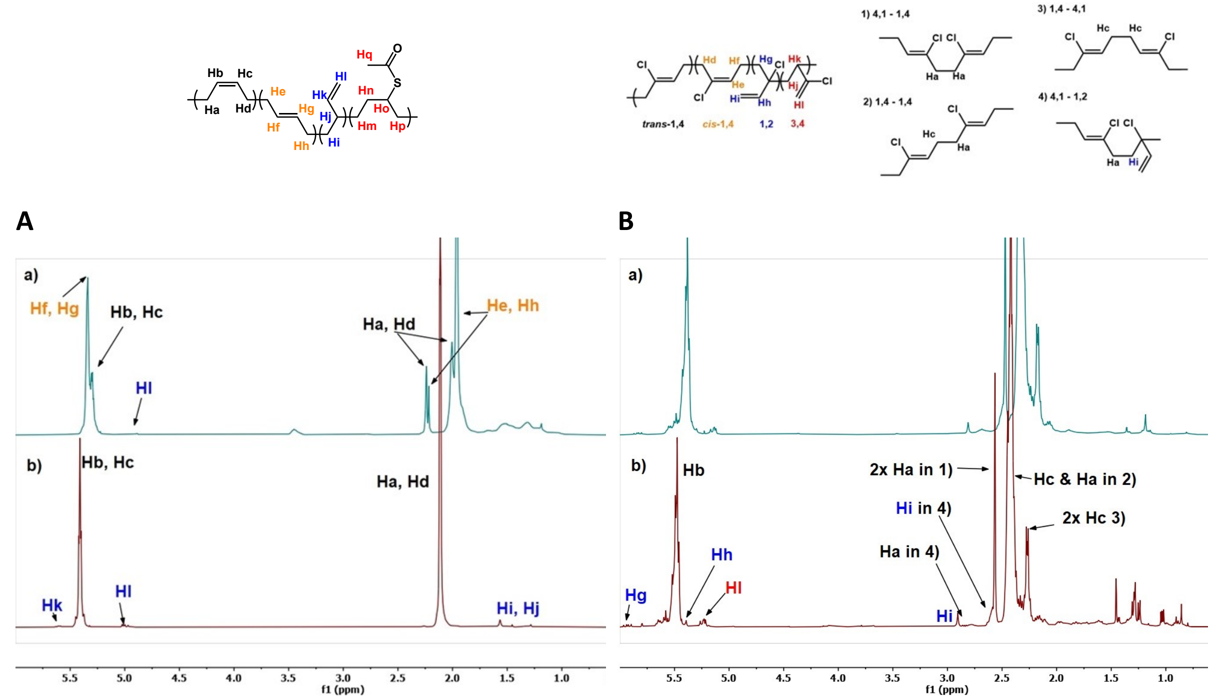

Figure S22 BR/TAA structure for ^1H NMR interpretation & ^1H NMR 400 MHz in $CDCl_3$ comparison between **a)** BR/TAA5% and **b)** BR; (**B**) CR structure for ^1H NMR interpretation and different unit arrangement &^1H NMR 400 MHz in $CDCl_3$ comparison between **a)** CR/TAA5% and **b)** CR;

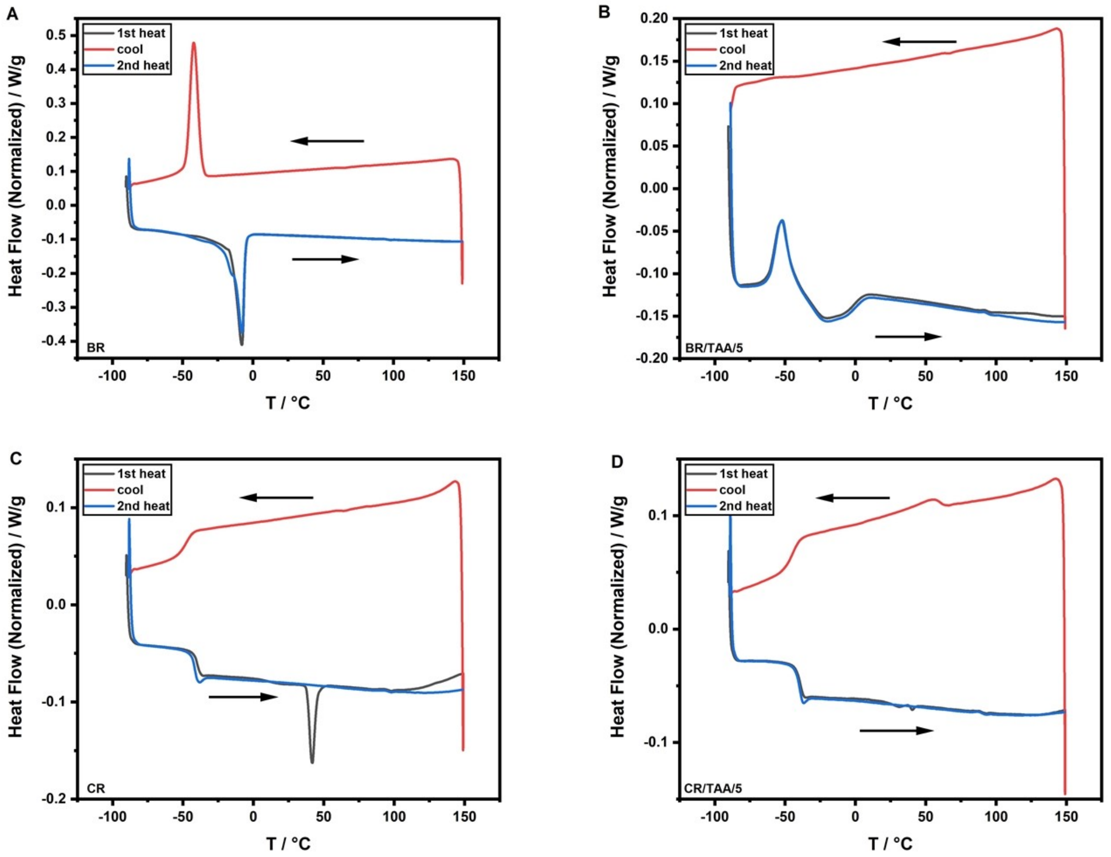

Figure S23 Dynamic DSC, heat-cool-heat experiments with heating and cooling ramps of 5°C/min; comparison between (**A**) BR & (**B**) BR/TAA/5%, and between (**C**) CR & (**D**) CR/TAA/5%. Exo up ↑.
